# Genetic Variations in *TNFAIP3* and *CTLA4* and Their Association with Circulating TNF-α and sCTLA4 Levels in Kurdish Patients with Rheumatoid Arthritis

**DOI:** 10.3390/genes17070821

**Published:** 2026-07-18

**Authors:** Sonia Elia Ishaq, Taban Kamal Rasheed, Niaz Albarzinji, Shukur Wasman Smail

**Affiliations:** 1 Department of Biology, College of Science, Salahaddin University-Erbil, Erbil 44001, Kurdistan Region, Iraq; taban.rasheed@su.edu.krd; 2 College of Medicine, Hawler Medical University, Erbil 44001, Kurdistan Region, Iraq; niazjawad@yahoo.com; 3 College of Pharmacy, Cihan University-Erbil, Erbil 44001, Kurdistan Region, Iraq; 4 Department of Medical Science, Respiratory Medicine, and Allergology, Respiratory Allergy and Sleep Research, Uppsala University and University Hospital, 75185 Uppsala, Sweden; 5 Department of Genetics, Bio Diagnostic Center (BDC), Erbil 44001, Kurdistan Region, Iraq

**Keywords:** rheumatoid arthritis, *TNFAIP3*, *CTLA4*, TNF-α, sCTLA-4

## Abstract

**Background/Objectives**: Rheumatoid arthritis (RA) is a chronic autoimmune disease marked by sustained synovial inflammation and systemic immune activation. Genetic polymorphisms in key immune-regulatory pathways are thought to influence both disease susceptibility and progression. This study aimed to characterize sequence variation within selected coding regions of *TNFAIP3* and *CTLA4* in Kurdish patients with RA and to evaluate circulating TNF-α and soluble CTLA-4 (sCTLA-4) levels in the same cohort. **Methods:** Eighty-seven participants (67 RA patients, 20 controls) were enrolled. Serum TNF-α and sCTLA-4 concentrations were quantified by ELISA. Genomic DNA was extracted and targeted Sanger sequencing of selected coding regions of *TNFAIP3* and *CTLA4* was performed in RA patients. Variant identification was conducted relative to GRCh38 and annotated using dbSNP and Genome Aggregation Database (gnomAD). GeneMANIA was used for network-based contextualization. **Results:** In the 218-bp targeted region of *TNFAIP3*, 103 sequence-level variant occurrences were identified in RA patients, including substitutions and insertion/deletion events. In the 136-bp targeted region of CTLA4, 33 sequence-level variant occurrences were identified, also including substitutions and insertion/deletion events. RA patients exhibited significantly elevated serum TNF-α levels compared with controls (*p* = 0.025), whereas sCTLA-4 levels did not differ significantly. **Conclusions:** This study provides the first targeted characterization of *TNFAIP3* and *CTLA4* genetic variation in Kurdish RA patients and integrates these findings with circulating inflammatory biomarkers. Although not designed to assess disease susceptibility, the study provides a valuable descriptive genetic resource for this population and complements existing evidence implicating NF-κB regulation and immune checkpoint signaling in RA. These findings establish a strong foundation for future case–control, genotype–phenotype, and functional investigations.

## 1. Introduction

Rheumatoid arthritis (RA) is a chronic inflammatory disease that affects joints, causes discomfort, and impairs function. It primarily affects small joints, although it can also affect other organ systems. Recent epidemiological data reveal that RA affects around 17.6 million people worldwide [1]. Although the etiology of RA is not yet fully understood, both genetic and environmental factors are crucial in the course of the disease. Up to 60% of the predisposition to RA has been attributed to genetic factors [2,3].

Many inflammatory mediators involved in the pathogenesis of RA, including TNF-α, IL-1, and IL-6, are markedly overexpressed in this condition and are regulated by nuclear factor kappa B (NF-κB). NF-κB also regulates the development of CD4+ T cells, namely activated T helper (Th)1, Th17, and auto-reactive CD4+ T cells, which play a crucial role in the pathophysiology of inflammation and autoimmune disease [4,5,6,7,8,9].

The ubiquitin-editing enzyme A20, encoded by the TNF-α-induced protein 3 (*TNFAIP3*) gene on chromosome 6, is a key negative regulator of NF-κB signaling. A20 plays a crucial role in immune cell differentiation, manages the production of inflammatory mediators, protects against autoimmunity, regulates NLRP3 inflammasome activity, and affects cell death and proliferation, thus acting as a tumor suppressor [10,11,12,13,14]. Structurally, A20 contains an N-terminal ovarian tumor (OTU) domain and seven C-terminal zinc finger (ZNF) domains. Since the first description of haploinsufficiency of A20 (HA20) in 2016, multiple frameshift and nonsense mutations affecting both domains have been reported [15,16]. Genetic alterations in *TNFAIP3*, including single-nucleotide polymorphisms (SNPs), have been associated with RA and other autoimmune diseases such as inflammatory bowel disease, systemic lupus erythematosus, type 1 diabetes, psoriasis, and coeliac disease [15,17]. Accordingly, targeted investigation of *TNFAIP3* in Kurdish RA patients may help define population-specific sequence diversity in an understudied group.

Cytotoxic T-lymphocyte-associated protein 4 (CTLA4), a recognized non-HLA susceptibility gene for RA, is expressed on regulatory and conventional T cells. CTLA 4 inhibits autoreactive T-cell responses by binding CD80/CD86 ligands with higher affinity than CD28, thereby delivering inhibitory signals [18,19]. Variants in *CTLA4* have been associated with multiple autoimmune disorders [20,21,22]. Because RA is a T-cell-mediated autoimmune disease and CTLA4 is a central immune checkpoint regulator, *CTLA4* variation remains biologically relevant to RA pathogenesis [18,20,23]. Accordingly, targeted analysis of *CTLA4* in Kurdish RA patients may help define population-specific sequence diversity relevant to immune dysregulation.

Despite extensive genetic investigations of RA in European and East Asian populations [24,25,26], data from Middle Eastern populations remain limited. The Kurdish population represents a genetically distinct and historically understudied group in immunogenetic research. Characterizing genetic variation in key immune-regulatory genes such as *TNFAIP3* and *CTLA4* within this population may provide valuable insight into population-specific immunogenetic architecture. Therefore, the present study aimed to characterize genetic variation within selected coding regions of *TNFAIP3* and *CTLA4* in Kurdish patients with RA and to integrate these findings with circulating inflammatory biomarkers, including TNF-α and soluble CTLA-4.

## 2. Methods

### 2.1. Patient Population

Sixty-seven RA patients were recruited from the rheumatology department at Rizgary Teaching Hospital and the outpatient clinic at CMC Private Hospital in Erbil. All patients met the 2010 RA classification criteria established by the American College of Rheumatology (ACR) and the European League Against Rheumatism (EULAR) [27]. Twenty healthy volunteers were recruited as control subjects. The study lasted nine months, from June 2024 to March 2025. The Human Ethics Committee of Salahaddin University approved the study (Approval No. 45/224; 7 May 2024). Each participant provided written informed consent.

The disease activity was measured based on the Disease Activity Score in 28 Joints (DAS28), which included the number of tender and swollen joints, the erythrocyte sedimentation rate (ESR), and the overall assessment by the patient [28].

Participants with additional autoimmune disorders, serious infections, or cancers, or those who were pregnant, were not included in the study. Healthy participants were required to have no history of autoimmune, chronic infectious, or inflammatory diseases.

### 2.2. Blood Sample Collection

Five millilitres of peripheral blood were drawn from all participants. Two millilitres of blood were placed in an EDTA tube for full blood count analysis and genomic DNA extraction, while the remaining three millilitres were transferred to a clot activator tube to separate the serum. After centrifugation at 3000 rpm for 10 min, the serum was carefully taken out and stored in an Eppendorf tube at −70 °C for later testing of TNF-α, sCTLA-4, and autoantibody levels.

### 2.3. Determination of Serum TNF-α and Soluble CTLA-4

Serum TNF-α and sCTLA-4 concentrations were measured using a sandwich enzyme-linked immunosorbent assay (ELISA), following the instructions provided by the manufacturer (Wuhan Feiyue Biotechnology Co., Ltd., Wuhan, China).

### 2.4. Measurement of RF and Anti-CCP Antibodies

The rheumatoid factor (RF) was quantified by the Roche Cobas 6000 analyser (Roche, Penzberg, Germany), with a positivity threshold of 14 IU/mL and a detection limit of 7 IU/mL. Anti-cyclic citrullinated peptide (anti-CCP) antibodies were assessed using electrochemiluminescence immunoassay (ECLIA), with a positive threshold set at 17 U/mL and a detection limit of 8 U/mL. All procedures adhered to the manufacturer’s specifications.

### 2.5. DNA Extraction, PCR Amplification, and Sanger Sequencing

Genomic DNA was extracted and purified from blood samples obtained from RA patients using the AddPrep Genomic DNA Extraction Kit (Addbio, Gyeongbuk, Republic of Korea), in accordance with the manufacturer’s guidelines. DNA was quantified using the Thermo Scientific NanoDrop 1000 spectrophotometer, and DNA purity was assessed using the A260/A280 absorbance ratio, with values of approximately 1.8 considered acceptable.

We analyzed a specific region of the *TNFAIP3* and *CTLA4* genes. The primers used were as follows: for *TNFAIP3*, forward primer GTA GGG CTG GTT TAT TCT G, and reverse primer TAA AGT TGC GTG TGT CTG; for *CTLA4*, forward primer CCT GAA GAC CTG AAC ACC G, and reverse primer CAG AAG ACA GGG ATG AAG AGA AG.

These amplicons were selected as part of a targeted candidate-region strategy to sequence coding regions of *TNFAIP3* and *CTLA4* because of the established roles of these genes in immune regulation and RA, rather than to achieve full-gene coverage. The reported PCR product sizes correspond to the complete amplified fragments, whereas the aligned sequence lengths reflect the high-confidence trimmed regions used for final alignment and variant calling. Low-quality terminal bases and primer-adjacent sequences were excluded when necessary to ensure analytical accuracy.

A ready-to-use master mix (AMPLIQON, Odense, Denmark) was used, which contains Taq DNA polymerase, dNTPs, KCl, and reaction buffer. The following PCR thermocycling parameters were used: an initial denaturation at 95 °C for 4 min, followed by 35 cycles of 94 °C for 40 s, annealing at 55 °C for 55 s for *TNFAIP3* and 61 °C for 40 s for *CTLA4*, and elongation at 72 °C for 45 s, all culminating in a final extension at 72 °C for 7 min.

PCR results were resolved using 2% agarose gel electrophoresis and analyzed with a 100 bp DNA marker (Pishgam, Isfahan, Iran). A 100-bp DNA ladder containing fragments of known sizes was used to estimate the sizes of the PCR products. Prior to casting onto the tray, the gel was stained with Safe dye (DNA Gel Staining solution; Pishgam, Iran). A gel documentation device (UV light Transilluminator UST-20M-8K; Biostep GmbH, Jahnsdorf, Germany) was used for gel visualization.

The product was sent to Macrogen, South Korea, following the PCR procedure, where it was analyzed on an automated 3130 genetic analyzer (Applied Biosystems; Thermo Fisher Scientific, Inc., Waltham, MA, USA). The same forward and reverse primers were used for each specified region of the gene.

All sequencing chromatograms were evaluated using bidirectional reads generated from forward and reverse primers. Variant calls were retained only when supported by clear chromatogram peak separation and consistent signal patterns in both sequencing directions. Base positions showing ambiguous peaks were excluded from analysis. Insertions and deletions were further verified by manual inspection of chromatogram peak shifts to minimize potential sequencing or alignment artifacts.

### 2.6. Variant Annotation and Bioinformatic Analysis

Sequence alignment and variant identification were performed relative to the human reference genome GRCh38 (hg38) using the NCBI Genome Data Viewer. Variants were annotated using dbSNP and Genome Aggregation Database (gnomAD) to determine whether they had previously been catalogued and to obtain available population-frequency information. A variant was classified as novel only when no matching record was found in dbSNP at the corresponding GRCh38 genomic position within the sequenced region. Sequence data have been deposited in the GenBank database (http://www.ncbi.nlm.nih.gov/genbank/) (accessed on 22 June 2026). The nucleotide sequence data are available in the GenBank database under the accession numbers PV740668.1 https://www.ncbi.nlm.nih.gov/nuccore/PV740668.1?report=genbank (accessed on 22 June 2026) and PV740669.1 https://www.ncbi.nlm.nih.gov/nuccore/PV740669.1/. (accessed on 22 June 2026).

The gnomAD was used to contextualize identified variants within global population reference data. In this study, gnomAD served as an annotation resource rather than as a primary outcome measure. Gene interactions between *TNFAIP3* and *CTLA4* were explored using the GeneMANIA prediction tool. This analysis was used as an exploratory network-context tool and not as experimental validation of the observed sequence variation.

### 2.7. Statistical Analysis

All statistical analyses were performed using GraphPad Prism version 9.0. The normality of continuous variables was assessed using the Shapiro–Wilk test. Age was expressed as mean ± standard deviation and compared between groups using Welch’s independent-samples *t*-test. Non-normally distributed variables were presented as median and interquartile range and compared using the Mann–Whitney U test. Categorical variables were expressed as numbers and percentages, and sex distribution was compared using Fisher’s exact test because of the small expected cell counts. All statistical tests were two-sided, and a *p* value < 0.05 was considered statistically significant.

## 3. Results

### 3.1. Participant Demographics and Clinical Characteristics

The present study enrolled 87 participants, of whom 67 were diagnosed with RA, while the remaining 20 served as healthy controls. Among the patients with RA, 93% were female and 7% were male, with a mean age of 55.79 ± 11.07 years. Eighty percent of the controls were female and 20% were male, with a mean age of 50.50 ± 11.63 years. No significant differences in age or gender were detected between the two groups. Among the RA group, 36 patients tested positive for RF, and 26 were positive for anti-CCP antibodies. The median DAS28-ESR was 4.605 (range: 1.680–7.600). Further information concerning family history of RA, disease duration, and smoking status is presented in Table 1.

### 3.2. Circulating TNF-α and sCTLA-4 Levels in RA Patients and Controls

**Figure 1:**
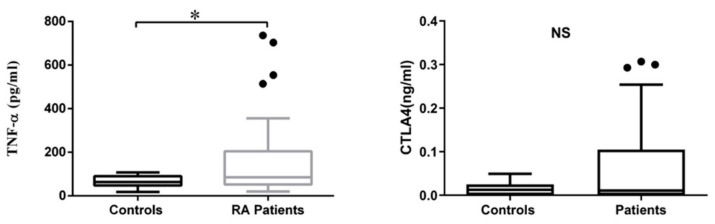
Comparison of serum TNF-α and sCTLA-4 concentrations between patients with rheumatoid arthritis and healthy controls using the Mann–Whitney U test. An asterisk indicates statistical significance at *p* < 0.05, whereas NS indicates no statistically significant difference. Abbreviations: RA, rheumatoid arthritis; TNF-α, tumor necrosis factor alpha; sCTLA-4, soluble cytotoxic T-lymphocyte-associated protein 4; NS, not significant.

Serum levels of TNF-α were considerably higher in patients with RA compared to healthy controls (*p* value = 0.025), indicating increased systemic inflammation in the RA group. Although serum concentrations of sCTLA-4 were higher in RA patients than in controls, this difference was not statistically significant (*p* value = 0.214) (Table 2, Figure 1).

### 3.3. Genetic Variant Analysis of TNFAIP3 and CTLA4

**Figure 2:**
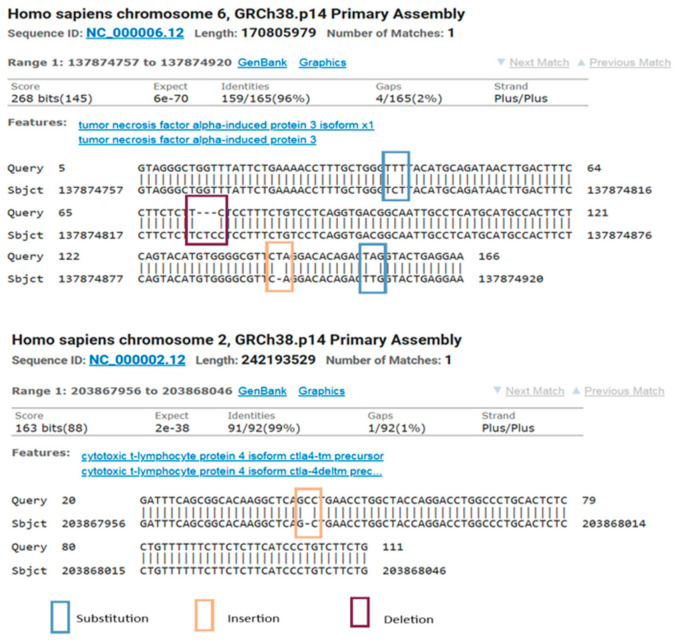
Representative NCBI BLAST sequence alignments showing variants identified in *TNFAIP3* and *CTLA4* in patients with rheumatoid arthritis. Patient-derived nucleotide sequences were aligned with the corresponding GRCh38 reference sequences. Colored boxes indicate substitutions, insertions, and deletions. BLAST, Basic Local Alignment Search Tool; NCBI, National Center for Biotechnology Information; GRCh38, Genome Reference Consortium Human Build 38; RA, rheumatoid arthritis; *TNFAIP3*, tumor necrosis factor alpha-induced protein 3; *CTLA4*, cytotoxic T-lymphocyte-associated protein 4.

Genomic DNA was isolated from peripheral blood samples collected from Kurdish individuals diagnosed with RA and residing in the Kurdistan region of Iraq. Representative alignment outputs confirming the *TNFAIP3* and *CTLA4* sequence variants are shown in Figure 2.

Across the 218-bp sequenced region of *TNFAIP3*, 103 sequence-level variant occurrences were identified in the RA cohort. These comprised 82 substitutions, 15 deletions, and 6 insertions. Of the 82 substitutions, 56 were novel SNVs and 26 matched previously reported dbSNP-annotated SNVs. At the functional annotation level, the variants were classified as 5 synonymous substitutions, 15 missense substitutions, and 1 nonsense substitution, whereas insertions and deletions are summarized in Table 3 as 5 deletions and 2 insertions with frameshift/disruptive effects.

**Figure 3:**
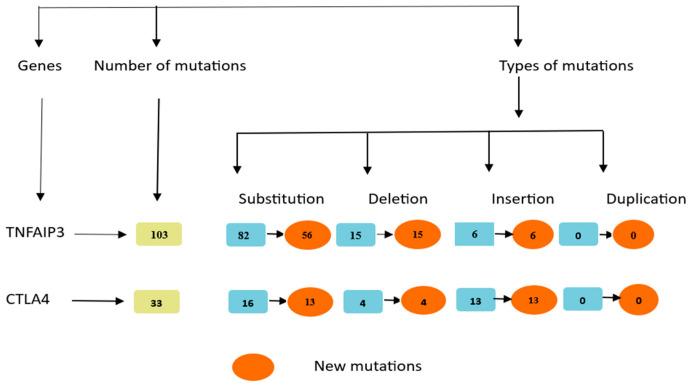
Flowchart summarizing sequence-level variants identified in *TNFAIP3* and *CTLA4* in patients with rheumatoid arthritis using the NCBI Genome Data Viewer. The total numbers of variant occurrences and their classification as substitutions, deletions, insertions, or duplications are shown for each gene. Yellow boxes represent the total number of sequence-level variant occurrences detected in each gene, blue boxes represent the number of variants within each mutation category, and orange ovals represent novel variants. Arrows indicate the classification workflow from total variants to individual mutation categories. RA, rheumatoid arthritis; NCBI, National Center for Biotechnology Information; *TNFAIP3*, tumor necrosis factor alpha-induced protein 3; *CTLA4*, cytotoxic T-lymphocyte-associated protein 4.

Figure 3 summarizes sequence-level variant occurrences, whereas Table 3 summarizes the corresponding functional annotations. Sequence-level mutations occurring downstream of a frameshift, including substitutions, insertions, and deletions, were not counted as separate amino acid consequences because the reading frame had already been disrupted.

Across the 136-bp sequenced region of *CTLA4*, 33 sequence-level variant occurrences were identified. These comprised 16 substitutions, 4 deletions, and 13 insertions. Of the 16 substitutions, 13 were novel SNVs and 3 matched previously reported dbSNP-annotated SNVs (Figure 3). At the functional annotation level, the variants were classified as 2 synonymous substitutions and 8 missense substitutions, whereas insertions and deletions were summarized in Table 3 as 3 deletions and 7 insertions with frameshift/disruptive effects.

Detailed variant annotations for both genes, including chromosome, genomic position, dbSNP identifier (rsID), reference and alternate alleles, HGVS genomic notation, variant type, allele frequency, and predicted functional consequence, are provided in Supplementary Tables S1 and S2.

Table 3 summarizes the unique predicted functional consequences of the sequence-level variants identified in *TNFAIP3* and *CTLA4*. The total numbers of sequence-level variant occurrences are presented separately in Figure 3. Variants occurring downstream of a frameshift event, including substitutions, insertions, and deletions, were not counted as separate amino acid consequences because the reading frame had already been disrupted. Accordingly, the totals shown in this table represent unique functionally annotated variants rather than all sequence-level variant occurrences. AA, amino acid; RA, rheumatoid arthritis; *CTLA4*, cytotoxic T-lymphocyte-associated protein 4; *TNFAIP3*, tumor necrosis factor alpha-induced protein 3.

### 3.4. Database-Derived Mutations and Gene Interactions

**Figure 4:**
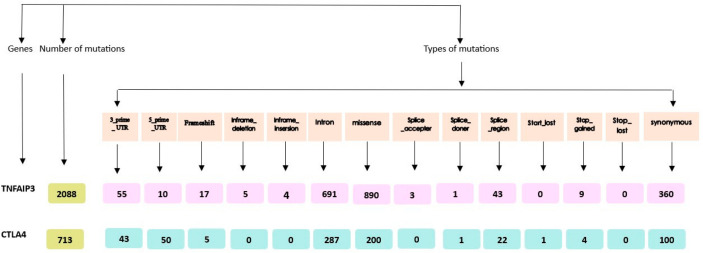
Flowchart summarizing the numbers and predicted functional consequences of genetic variants retrieved from the Genome Aggregation Database for *TNFAIP3* and *CTLA4*. A total of 2088 variants were listed for *TNFAIP3* and 713 variants for *CTLA4*. Variants were categorized according to their annotated consequences, including untranslated-region, frameshift, in-frame, intronic, missense, splice-site, start-loss, stop-gain, stop-loss, and synonymous variants. Yellow boxes represent the total number of variants reported for each gene, whereas the colored boxes represent the number of variants within each functional consequence category. Arrows indicate the classification workflow from the total variant count to the individual consequence categories. These database-derived data were used for contextual comparison and were not considered direct outcomes of the present study. gnomAD, Genome Aggregation Database; UTR, untranslated region; *TNFAIP3*, tumor necrosis factor alpha-induced protein 3; *CTLA4*, cytotoxic T-lymphocyte-associated protein 4.

The queried gnomAD dataset listed 2088 variants in *TNFAIP3* and 713 variants in *CTLA4* (Table S3 and Figure 4). These data were used to provide population context for interpretation of the sequenced variants, not as direct study outcomes.

**Figure 5:**
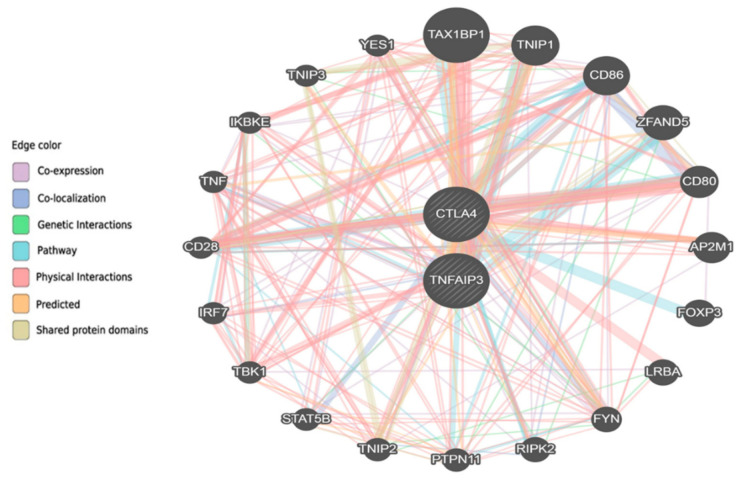
GeneMANIA interaction network showing *TNFAIP3*, *CTLA4*, and their associated genes. Colored edges represent different types of relationships, including co-expression, co-localization, genetic interactions, pathway associations, physical interactions, predicted interactions, and shared protein domains. This network analysis was used for exploratory and hypothesis-generating purposes and does not provide experimental confirmation of functional interactions. GeneMANIA, Gene Multiple Association Network Integration Algorithm; *TNFAIP3*, tumor necrosis factor alpha-induced protein 3; *CTLA4*, cytotoxic T-lymphocyte-associated protein 4.

According to the GeneMANIA prediction tool, there are 13 genes (*CD80, FYN, IKBKE, IRF7, RIPK2, STAT5B, TAX1BP1, TBK1, TNF, TNIP1, TNIP2, TNIP3,* and *ZFAND5*) related to the *TNFAIP3* gene, and 11 genes (*AP2M1, CD28, CD80, CD86, FOXP3, FYN, IKBKE, LRBA, PTPN11, STAT5B,* and *YES1*) related to the *CTLA4* gene via co-expression, co-localization, genetic interaction, pathway, physical interaction, predicted, or shared protein domain (Table S4, Figure 5). The two networks shared four genes, *CD80, FYN, IKBKE, and STAT5B*, suggesting potential convergence within related immune-regulatory pathways. This network analysis is supportive and hypothesis-generating rather than confirmatory.

## 4. Discussion

This study provides a population-specific characterization of variants in *TNFAIP3* and *CTLA4* among Kurdish patients with RA, integrated with circulating inflammatory biomarker measurements. We observed extensive genetic variation in both genes together with markedly elevated serum TNF-α levels compared to healthy controls, while sCTLA-4 levels remained similar between groups. The study therefore defines a clinically grounded immunogenetic profile in an understudied population, while stopping short of claims about disease susceptibility or functional causality that were not directly tested.

*TNFAIP3* encodes the A20 protein, a ubiquitin-editing enzyme that serves as a critical negative regulator of NF-κB signaling by deubiquitinating intermediates such as RIPK1 and TRAF6. Pathogenic loss-of-function variants in *TNFAIP3* can impair this regulatory system, resulting in prolonged NF-κB activation, cytokine production, and chronic inflammation. Previous research has linked *TNFAIP3* polymorphisms and haploinsufficiency to autoimmune disorders, particularly early-onset and treatment-resistant RA characterized by heightened TNF-α levels and systemic immune activation [26,29,30,31]. The significant elevation of TNF-α observed in our cohort is consistent with the known regulatory role of A20 in limiting NF-κB-mediated inflammatory signaling. However, because functional assays were not performed, the identified variants should be interpreted as sequence findings with biologically plausible relevance rather than demonstrated mechanistic drivers.

In animal model studies, deletion of *TNFAIP3* in the myeloid cells results in spontaneous erosive polyarthritis, which is marked by chronic NF-κB activation, high levels of pro-inflammatory cytokines and high osteoclast activity. These data highlight the critical importance of A20 in the regulation of inflammation and bone homeostasis [32]. Likewise, Wang and colleagues provided the first report of reduced TNFAIP3 mRNA expression in peripheral blood mononuclear cells (PBMCs) of patients with RA. Using RT-qPCR in 48 RA patients and 41 age- and sex-matched healthy controls, they showed that TNFAIP3 transcript levels were significantly lower in RA than in controls (21.32 vs. 52.58; *p* = 0.0125) and were further reduced in anti-CCP-positive patients relative to anti-CCP-negative patients (19.44 vs. 27.67; *p* = 0.0134). TNFAIP3 expression correlated inversely with the ACR/EULAR RA classification score (r = −0.596), anti-CCP antibody titer (r = −0.622), and CRP (r = −0.591; all *p* = 0.001). The authors concluded that insufficient TNFAIP3 expression is associated with the diagnosis and severity of RA and proposed TNFAIP3 as a candidate target for gene- or pharmacology-based therapeutic intervention, while acknowledging that their study was limited to transcript quantification and did not include protein-level or mechanistic data [30]. Taken together, these studies support the biological relevance of TNFAIP3-mediated NF-κB regulation in RA, and our data extend that literature by defining the variant spectrum in Kurdish RA patients.

The *CTLA4* gene is located on chromosome 2q33 and encodes a 223-amino-acid receptor that inhibits T-cell activation. It does this by binding to the B7 ligands (CD80/CD86) with higher affinity than the costimulatory receptor CD28 [33]. Genetic variation in *CTLA4* has been associated with altered protein expression, structure, and function, contributing to RA susceptibility [22,34,35,36,37,38]. In vivo experiments show that CTLA4 intra-articular delivery or CTLA-4Ig can alleviate experimental arthritis and decrease pro-inflammatory cytokine production in RA synovial macrophages and T helper cells [39,40,41].

Cao and colleagues reported that RA patients exhibited elevated serum sCTLA-4 levels compared to healthy controls, with a positive correlation to DAS28 scores [42]. In our cohort, circulating sCTLA-4 levels were not significantly different between patients and controls, indicating that serum abundance alone does not capture the complexity of CTLA4-related immune regulation in RA.

The rs231775 (+49 A > G) polymorphism was detected in the sequenced region and is listed in Table S1. This variant has been reported to reduce CTLA4 inhibitory function in prior studies [20,43], and its presence in our cohort is consistent with previously reported *CTLA4* variation in autoimmune disease. Although these findings support the biological plausibility of this variant, the present study was designed to characterize sequence variation rather than genotype–phenotype relationships.

Accordingly, the Kurdish RA cohort exhibited a spectrum of TNFAIP3 and CTLA4 sequence variation together with elevated TNF-α levels and no statistically significant difference in circulating sCTLA-4 concentrations. These findings expand the molecular characterization of RA in the Kurdish population and provide a basis for future studies to determine whether the identified variants are associated with clinical characteristics, disease activity, or biomarker profiles.

## 5. Conclusions

This study provides a targeted characterization of selected coding regions of *TNFAIP3* and *CTLA4* in Kurdish patients with RA. By integrating targeted sequencing with circulating biomarker measurements, the findings highlight potential links between NF-κB regulation, immune checkpoint signaling, and inflammatory activity in RA. The dataset defines a population-specific immunogenetic profile and a biomarker baseline for this understudied population. Although the present study does not support direct claims of disease susceptibility, causality, or genotype-specific biomarker regulation, it establishes a solid foundation for larger-scale genetic, phenotype-linked, and functional investigations.

## 6. Limitations

The principal methodological boundaries of this study include sequencing restricted to predefined regions of *TNFAIP3* and *CTLA4*, the modest cohort size, and the absence of genetically sequenced healthy controls. In addition, treatment status may have influenced serum biomarker measurements, and no genotype–biomarker association analysis was performed in the current dataset. Although these factors constrain variant-level association analyses and limit generalizability, the study nonetheless identifies relevant genetic variation and integrates these findings with biomarker data in an understudied population. This work should therefore be interpreted as a population-specific targeted sequencing study with strong descriptive and hypothesis-generating value.

## Figures and Tables

**Table 1:** Demographic and clinical characteristics.

Variables	Controls *n* = 20	RA Patients *n* = 67	*p* Value
Age (mean ± SD)	50.50 ± 11.63	55.79 ± 11.07	0.081 ^a^
Sex, male/female, *n* (%)	4 (20%)/16 (80%)	5 (7%)/62 (93%)	0.202 ^b^
Smoking (%)	0	7	
Family history of RA (%)	—	52	
RA duration (years), median (range)	—	10.00 (1.000–35.00)	
RF (IU/mL) (Positive cases)	0	36	
Anti-CCP U/mL (Positive cases)	0	26	
DAS28-ESR, median (range)	—	4.605 (1.680–7.600)	

Data are presented as mean ± standard deviation, median (interquartile range), number (percentage), or percentage, as appropriate. Age was compared between groups using Welch’s independent-samples *t*-test. Sex distribution was compared using Fisher’s exact test because at least one expected cell count was less than 5. All tests were two-sided, and a *p* value < 0.05 was considered statistically significant. The dash (—) indicates that the variable was not applicable or was not compared between groups. Percentages may not total exactly 100% because of rounding. Anti-CCP, anti-cyclic citrullinated peptide antibody; DAS28-ESR, Disease Activity Score in 28 joints based on erythrocyte sedimentation rate; RA, rheumatoid arthritis; RF, rheumatoid factor; SD, standard deviation. ^a^ Welch’s independent-samples *t*-test. ^b^ Fisher’s exact test.

**Table 2:** Serum Levels of TNF-α and sCTLA-4.

Parameters	Controls *n* = 20 Median (Interquartile Range)	Patients *n* = 67 Median (Interquartile Range)	*p* Value
TNF-α (pg/mL)	63.64 (47.99–90.27)	85.23 (52.20–204.4)	0.025 *
sCTLA-4 (ng/mL)	0.013 (0.000–0.024)	0.060 (0.000–0.105)	0.214

Data are presented as median (interquartile range). Comparisons between patients with rheumatoid arthritis and healthy controls were performed using the two-sided Mann–Whitney U test. A *p* value < 0.05 was considered statistically significant. An asterisk (*) indicates a statistically significant difference. IQR, interquartile range; RA, rheumatoid arthritis; sCTLA-4, soluble cytotoxic T-lymphocyte-associated protein 4; TNF-α, tumor necrosis factor alpha; ng/mL, nanograms per millilitre; pg/mL, picograms per millilitre.

**Table 3:** Summary of unique functional annotations corresponding to sequence-level variant occurrences in *TNFAIP3* and *CTLA4* genes with RA.

Gene	Total Mutations	Total Unique Functionally Annotated Variants	Amino Acid Change Type	Count
*TNFAIP3*	28	Substitution	Synonymous (no AA change)	5
			Missense (AA change)	15
			Nonsense (premature stop)	1
		Deletion	Frameshift/disruptive	5
		Insertion	Frameshift/disruptive	2
*CTLA4*	20	Substitution	Synonymous (no AA change)	2
			Missense (AA change)	8
			Nonsense (premature stop)	0
		Deletion	Frameshift/disruptive	3
		Insertion	Frameshift/disruptive	7

## Data Availability

The data used and/or analyzed during the current study are available from the corresponding author on reasonable request.

## Supplementary Materials

The following supporting information can be downloaded at: https://www.mdpi.com/article/10.3390/genes17070821/s1, Table S1: Variants identified in TNFAIP3 and CTLA4 genes in rheuma-toid arthritis patients analyzed using DNA mutation variant analysis; Table S2: Detailed functional annotations corresponding to sequence-level variant occurrences in the TNFAIP3 and CTLA4 genes from rheumatoid ar-thritis patients; Table S3: Summary of mutations in the TNFAIP3 and CTLA4 genes retrieved from the Genome Aggregation Database (gnomAD); Table S4: Interactions between TNFAIP3 and CTLA4 and their related genes retrieved from the GeneMANIA database.

## Abbreviations

The following abbreviations are used in this manuscript:

ACR	American College of Rheumatology
Anti-CCP	Anti-cyclic citrullinated peptide antibodies
CTLA4	Cytotoxic T-lymphocyte-associated protein 4
DAS28	Disease Activity Score in 28 Joints
dbSNP	Database of Single Nucleotide Polymorphisms
EDTA	Ethylenediaminetetraacetic acid
ECLIA	Electrochemiluminescence immunoassay
ELISA	Enzyme-linked immunosorbent assay
ESR	Erythrocyte sedimentation rate
EULAR	European League Against Rheumatism
gnomAD	Genome Aggregation Database
GRCh38	Genome Reference Consortium Human Build 38
HA20	Haploinsufficiency of A20
HLA	Human leukocyte antigen
NCBI	National Center for Biotechnology Information
NF-κB	Nuclear factor kappa B
OTU	Ovarian tumor domain
PCR	Polymerase chain reaction
RA	Rheumatoid arthritis
RF	Rheumatoid factor
RT-qPCR	Reverse transcription quantitative polymerase chain reaction
sCTLA-4	Soluble cytotoxic T-lymphocyte-associated protein 4
SNP	Single-nucleotide polymorphism
SNV	Single-nucleotide variant
Th	T helper cell
TNF-α	Tumor necrosis factor-alpha
TNFAIP3	Tumor necrosis factor alpha-induced protein 3
ZNF	Zinc finger domain
